# Derivation and assessment of risk prediction models using case-cohort data

**DOI:** 10.1186/1471-2288-13-113

**Published:** 2013-09-13

**Authors:** Jean Sanderson, Simon G Thompson, Ian R White, Thor Aspelund, Lisa Pennells

**Affiliations:** 1 Department of Public Health and Primary Care, Strangeways Research Laboratory, University of Cambridge, Worts Causeway, Cambridge CB1 8RN, UK; 2 MRC Biostatistics Unit, Cambridge CB2 0SR, UK; 3 Center of Public Health Sciences, University of Iceland, Reykjavík 101, Iceland; 4 Icelandic Heart Association, Kopavogur 201, Iceland

**Keywords:** Case-cohort, Risk prediction, Discrimination, Reclassification, Cardiovascular disease

## Abstract

**Background:**

Case-cohort studies are increasingly used to quantify the association of novel factors with disease risk. Conventional measures of predictive ability need modification for this design. We show how Harrell’s C-index, Royston’s D, and the category-based and continuous versions of the net reclassification index (NRI) can be adapted.

**Methods:**

We simulated full cohort and case-cohort data, with sampling fractions ranging from 1% to 90%, using covariates from a cohort study of coronary heart disease, and two incidence rates. We then compared the accuracy and precision of the proposed risk prediction metrics.

**Results:**

The C-index and D must be weighted in order to obtain unbiased results. The NRI does not need modification, provided that the relevant non-subcohort cases are excluded from the calculation. The empirical standard errors across simulations were consistent with analytical standard errors for the C-index and D but not for the NRI. Good relative efficiency of the prediction metrics was observed in our examples, provided the sampling fraction was above 40% for the C-index, 60% for D, or 30% for the NRI. Stata code is made available.

**Conclusions:**

Case-cohort designs can be used to provide unbiased estimates of the C-index, D measure and NRI.

## Background

The case-cohort sampling design, initially introduced by Prentice [1], reduces the amount of data collection required compared to full cohort studies by efficient sampling of the non-diseased individuals. Information is collected on a random sample of the original cohort (the subcohort) as well as all additional cases. Together, the subcohort and the extra cases form the case-cohort set. Although follow-up for disease is still necessary for the full cohort, covariate information (which is often costly to obtain) is only required for the case-cohort set. Furthermore, if the subcohort is chosen at random (i.e. an unstratified design) then it can be used as a comparison group for multiple diseases of interest, offering an advantage over the nested case–control design. Hazard ratios for case-cohort studies are typically estimated using a weighted version of the Cox proportional hazards model [1]. Different weighting methods have been proposed and these methods are well documented in the literature [2]. Similarly, the baseline hazard can be estimated using a weighted version of the standard Breslow estimator, allowing estimation of absolute risks.

Although investigation of the bias in resulting hazard ratio estimates has been the subject of previous research [3], there has been little work on the accuracy and precision of risk predictions made using a model in which both the hazard ratios and baseline survival estimates have been derived using case-cohort data. Measures to quantify the predictive ability of risk models have been adapted and applied to case-cohort designs by Ganna et al [4] who describe the use of the C-index, reclassification and calibration for both stratified and unstratified case-cohort designs.

Time-dependent area under the ROC curve methods [5] have also been used in the case-cohort setting [6,7] and C-statistics and the net reclassification index (NRI) have been applied by Vaarhorst et al [8]. However, any resulting bias in the estimates and their standard errors (SEs) has not been extensively investigated and the use of other measures of predictive ability has so far, to our knowledge, not been considered. The use of predictive measures in case-cohort designs is of increasing relevance, as technological advances permit routine measurement of many novel biological and genomic markers for risk prediction. Large studies with various disease outcomes can be utilised efficiently by adopting a case-cohort design [9].

In this paper we describe methods for deriving risk prediction models from case-cohort data and focus on relevant adaptation of commonly used measures to assess their predictive ability: Harrell’s C [10,11], Royston and Sauerbrei’s D [12], and the NRI [13,14]. The proposed methods are extensively assessed through simulation, in which estimates from case-cohort data are compared to those which would have been seen in a full cohort study.

## Methods and results

### Data and simulations used

Our methods are illustrated using case-cohort samples which have been randomly sampled from a full cohort, and risk prediction models for coronary heart disease (CHD, defined as first ever non-fatal myocardial infarction or coronary death). For the full cohort we use the 6773 males from the Reykjavik prospective cohort study [15], yielding 1827 events from 153,428 person-years of follow-up (median follow-up was 24.5 years). Deaths from causes other than CHD are regarded as censoring events; we return to this point in the Discussion. Descriptive statistics and hazard ratios of CHD for the key covariates measured at study entry are provided in Table 1. Our initial examples use risk models including age, smoking, systolic blood pressure and total cholesterol (model 1 of Table 1). The NRI is assessed considering the addition of HDL-cholesterol to this model (model 2 of Table 1).

**Table 1:** Descriptive statistics and hazard ratios of CHD in the Reykjavik prospective cohort study

		Hazard ratio (95% CI) for CHD per 1 standard deviation higher value or in comparison with reference category*	
	**Mean (SD) or n (%)**	**Model 1**	**Model 2**
Age at survey (years)	52.3 (7.6)	1.68 (1.60, 1.77)	1.65 (1.57, 1.74)
Smoking status (smoker vs non-smoker)*	3,609 (53.3)	1.54 (1.40, 1.70)	1.51 (1.37, 1.66)
Systolic blood pressure (mmHg)	141.8 (20.6)	1.22 (1.17, 1.28)	1.22 (1.17, 1.28)
Total cholesterol (mmol/l)	6.3 (1.0)	1.31 (1.26, 1.37)	1.27 (1.22, 1.33)
HDL-cholesterol (mmol/l)	1.4 (0.2)		0.80 (0.76, 0.85)

Hazard ratios are based on a Cox proportional hazards model involving 6773 men (1827 events) with information on all risk factors, and using time from study entry as the time scale.

*reference category for smoking is non-smokers.

To encompass the variability that might be seen in practice, new outcome data were generated for the full cohort using original covariate values and observed parameters from a fitted Weibull model. New event times (using time from study entry as the time scale) were simulated following

(1) $$T_{i}={\left(-\log\left(U_{i}\right)\right)}^{\frac{1}{\hat{\gamma }}}\exp\left(\hat{\beta }x_{i}\right)$$

where $\hat{\beta }x_{i}$ is the linear predictor of each individual, $\hat{\gamma }$ is the Weibull shape parameter, and *U*_*i*_ is a randomly generated Uniform (0,1) variable. Staggered entry was imposed, permitting individuals to enter the study randomly between 0 and 5 years, and event times were censored at 25 years.

Our simulation procedure, designed to test the effectiveness of case-cohort methods compared to those utilising the full cohort, is as follows: 1) A new realisation of the full cohort is generated by simulating new outcome data as described above. In the simulated full cohort we derive a CHD risk prediction model, and calculate subsequent measures of predictive ability, thereby providing a “gold standard” to which results achieved with case-cohort samples can be compared. 2) A random subsample of the full cohort, with proportion α, and all the CHD cases, are selected to form the case-cohort set. The prediction model and appropriate measures of interest are then calculated in this subsample using case-cohort methods (described in later sections). This procedure (both steps 1 and 2) is repeated 1000 times for each of 18 choices of sampling fraction, α, with values 0.01, 0.02, … 0.1, 0.2, … 0.9.

### Deriving a risk prediction model using case-cohort data

We assume use of the Cox proportional hazards (CPH) model [16] as the risk prediction model. For full cohort data this takes the form

(2) $$S\left(t|x\right)=S_{0}{\left(t\right)}^{\exp\left(\mathrm{\beta x}\right)}$$

where t represents time from study entry, S(*t*|*x*) is the probability of surviving beyond time *t* given covariates *x* and *S*_0_(*t*) is the baseline survival function at time *t*. The vector **β** = (*β*_1_, *β*_2_, ⋯, *β*_*p*_) represents the multivariable adjusted log hazard ratios (HRs) per unit increase in the risk predictors $x_{i}^{T}={\left(x_{i1},x_{i2},\cdots ,x_{\mathrm{ip}}\right)}^{T}$. An individual’s estimated linear predictor is $\hat{\beta }x_{i}$$=\sum_{j}{\hat{\beta }}_{j}x_{\mathrm{ij}}$ and their estimated absolute risk of experiencing an event by time *t* is 1– ${\hat{S}}_{0}{\left(t\right)}^{\exp\left(\hat{\beta }x_{i}\right)}$. Due to the over-representation of cases in the case-cohort design, two adaptations are needed in risk model derivation as follows.

#### Estimating hazard ratios from case-cohort data

With case-cohort data a pseudolikelihood is used to estimate $\hat{\beta }$ from a weighted Cox regression, with cases outside the subcohort not considered ‘at risk’ until immediately before their event. Three commonly used weighting methods have been proposed: Prentice [1], Self & Prentice [17], and Barlow [2]. The Prentice and Self & Prentice methods differ only in the contribution of a non-subcohort case at the time of their event: with the Self & Prentice formulation only subcohort individuals are included in the risk set. The Barlow weighting scheme takes a different approach, weighting the subcohort controls and future cases by the inverse of the subcohort sampling fraction. This aims to mimic the proportions that would have been present in the full cohort analysis. These weighting methods have been previously described in detail and compared by simulation. The methods were shown to provide similar results with the Prentice method generally being preferred for small subcohort sizes [3].

The usual SE estimates for the CPH model are not valid in the case-cohort situation. A robust jackknife estimator was proposed [2] and can be implemented in most standard software. (Although the SE itself is not necessary for risk estimation, it may be influential in choice of risk predictors to include in the risk model).

#### Estimating baseline hazard from case-cohort data

In order to provide absolute risk predictions it is necessary to estimate the baseline survival function, *S*_0_(*t*). For case-cohort data this is achieved via a weighted version of the Breslow estimator of the cumulative baseline hazard [17,18]. This additional weighting applies to the Prentice and Self & Prentice weighting methods but not Barlow. The Barlow method mimics the proportions observed in the full cohort sample and so can be used for the estimation of absolute risk without any further rescaling [19].

#### Example: Absolute predictions using models derived with case-cohort data

**Figure 1:**
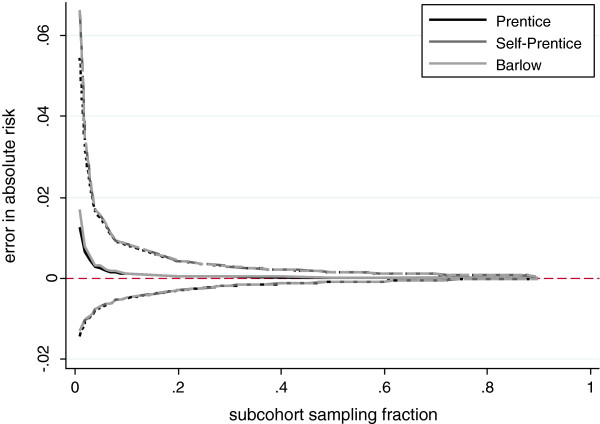
**Mean error in 10-year predicted absolute risk.** Figure shows the difference between risk predictions obtained using a prediction model derived on the case-cohort set, and those obtained using the model derived on the full cohort (case-cohort minus full cohort estimates). Solid lines show mean error, averaged over all individuals in the case-cohort set, and averaged over simulations, for three case-cohort weighting methods. Dashed lines show upper and lower fifth percentiles of the distribution of average differences across 1000 simulations.

The mean error in 10-year absolute risk predictions, as estimated using the three case-cohort weighting methods, in comparison to the full cohort estimates, is summarised in Figure 1 for a range of sampling fractions. For each individual in the case-cohort set, the risk predictions are produced using the model derived on the full cohort data and the model derived on the case-cohort set. The difference between the risk predictions is averaged over all individuals in the case-cohort sample.

On average the absolute risk tends to be overestimated at low subcohort sampling fractions, and the variability at low subcohort sampling fractions is also greater. Overall the three weighting methods provide very similar estimates of absolute risk (as evidenced by overlapping lines in Figure 1), although at very low sampling fractions the Prentice method is slightly more accurate. This is reflected by slightly more accurate estimates in both the baseline hazard and the log hazard ratios (Additional file 1: Figure S1 and Additional file 1: Figure S2) and concurs with previous findings [3]. Since the different weighting methods show little difference in terms of the resulting risk predictions, further results are presented based on the Prentice scheme only.

### Measures of predictive ability

We describe three measures of predictive ability: Harrell’s C-index [10,11], Royston and Sauerbrei’s D [12], and the net reclassification index (NRI) [13,14]. Although many other measures exist [20-22], these were selected because of their relevance and familiarity for the intended clinical and epidemiological audience. Adaptations for case-cohort data deal with the over-representation of cases compared to the original cohort design. Application of standard measures of predictive ability without considering the sampling of the data will result in bias.

#### C-index for case-cohort designs

The C-index estimates the probability that the predicted order of events is correct in a randomly selected pair of participants. It is estimated by examining all “relevant” pairs of participants for which the participant with the shorter participation time has an observed event. Pairs are classed as concordant *(n*_*c*_), discordant (*n*_*d*_) or undecided (*n*_*u*_) according to whether they agree, disagree or tie respectively, in rank of predicted risk and order of events. Since the predicted survival in (2) is a monotonic transformation of the baseline survival, $\hat{\beta }x_{i}$ can replace predicted risk. The C-index is calculated as:

(3) $$C=\frac{n_{c}+0.5n_{u}}{n_{r}},$$

where *n*_*r*_ = *n*_*c*_ + *n*_*d*_ + *n*_*u*_ is the total number of relevant pairs. A C-index value of 0.5 indicates no discriminative ability, beyond that of chance, and 1 indicates perfect discrimination.

To allow for the case-cohort sampling we present a weighted version of the standard measure, as previously proposed [4]. Informative pairs are divided into case-case pairs and case–control pairs. A weight of 1/α is applied to all case–control pairs, to allow for the fact that these pairings will be under-represented in the case-cohort design. Note that all cases are handled the same, irrespective of whether they are from the subcohort. Using a second subscript to denote whether the pairs are from case-case comparisons (1) or case–control comparisons (0), the weighted C measure is given by:

(4) $$C_{W}=\frac{n_{c,1}+0.5n_{u,1}+\frac{1}{\alpha }n_{c,0}+\frac{1}{\alpha }0.5n_{u,0}}{n_{r,1}+\frac{1}{\alpha }n_{r,0}}$$

where *n*_*i*,*j*_ represents the number of pairs in group *i* = concordant, discordant or uncertain, and *j* = 0 (case–control comparisons) or 1 (case-case comparisons). This can be implemented in Stata using the somersd [23] function with importance weights, which also gives an appropriately weighted standard error.

#### D for case-cohort designs

With full cohort data, Royston and Sauerbrei’s D is calculated by firstly transforming linear predictors ($\hat{\beta }x_{i}$) to give standard normal order rank statistics (using Blom’s approximation). The rank statistics are multiplied by $\sqrt{\pi /8}$ to give *z*_*i*_ for *i* = 1…*n* subjects. A second CPH model is then fitted to these values and the value of *D* is given by the coefficient of z in this second model. The distribution of z is approximately *N*(0, *π*/8) and has the property that the mean of the negative and positive z values are −0.5 and 0.5. *D* can therefore be interpreted as the log hazard ratio between the participants at higher risk versus those at lower risk. The SE of *D* is given by the SE of the coefficient of z.

In order to estimate *D* in the case-cohort design, we propose a novel weighted *D* measure. We assume $\hat{\beta }$ has been estimated using the weighted Cox regression described earlier, and apply the following adaptations:

1) Since controls are under-represented in the case-cohort set, the normal order rank statistics are formed using a modification that emulates the values that would have been obtained using the full cohort data: i) the “weighted ranks” are formed by applying an increased weight of 1/α to the subcohort controls; ii) the weighted normal order rank statistics are formed using Blom’s approximation, where the sample size is taken to be the maximum weighted rank (approximating the number in the full cohort); iii) as with the full cohort method, the weighted normal order rank statistics are then multiplied by $\sqrt{\pi /8}$ to give $z_{i}^{C}$.

2) A *weighted* CPH model is fitted to the $z_{i}^{C}$ (using any of the three weighting methods described earlier). Our case-cohort weighted estimate, *D*_*W*_, is given by the coefficient of *z*^*C*^ in this model and its SE is given as the SE of the coefficient of *z*^*C*^. As with estimating the hazard ratios, a robust variance estimator should be specified to obtain the appropriate SEs.

#### Example: Measures of discrimination calculated using case-cohort data

**Figure 2:**
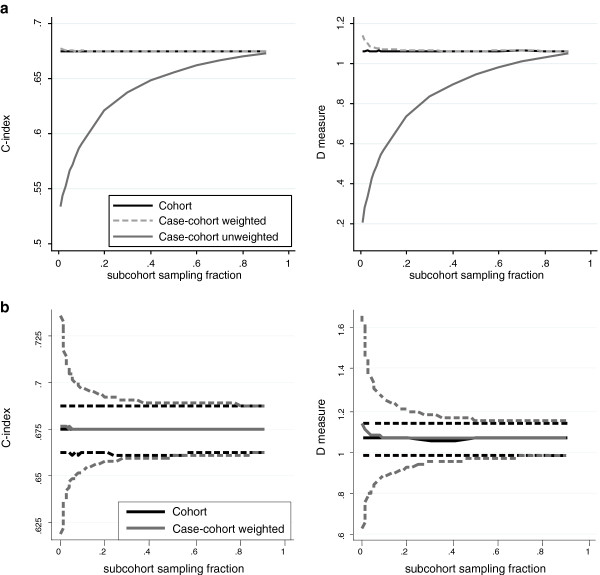
**Unweighted and weighted versions of Harrell’s C-index and Royston and Sauerbrei’s D measure.** Predictions are formed using a model in which the Prentice weighting scheme is applied. Graphs show the mean over 1000 selections of the subcohort at each sampling fraction for **a)** the standard unweighted methods and the weighted versions, and **b)** the proposed weighted versions with 95% confidence intervals calculated using the empirical standard errors.

Application of the C-index or D measure to case-cohort data, without any modification for the sampling design, results in biased estimates of concordance when compared to those using the full cohort data set (Figure 2a), estimated discrimination being substantially lower at small subcohort sampling fractions where the proportion of events is higher. In Figure 2a, the same case-cohort weighted estimates of $\hat{\beta }x_{i}$ are used for both the weighted and unweighted measures. The proposed adaptations to the C-index and D measure give results comparable to that which is seen with full cohort data.

Figure 2b shows the weighted case-cohort estimates only, along with the empirical 95% CI. As would be expected, the CI width of the adapted measures is greater at lower sampling fractions, and approaches that of the full cohort estimate as subcohort sampling fraction increases. The mean analytical SE agrees well with the empirical SE (over the 1000 simulations at each subcohort sampling fraction) as shown in Additional file 1: Figure S3.

#### Net reclassification improvement (NRI) for case-cohort designs

The NRI aims to assess whether the addition of a new factor to the risk prediction model leads to improvement in classification of participants into clinically relevant risk categories [13]. For each model (with and without the new factor of interest), absolute risk predictions are used to assign individuals to predefined risk categories. The NRI is calculated by considering movement between categories on addition of the new factor. Movements are considered separately for cases and non-cases based on event status at *t* years. The movement is deemed appropriate if cases move into a higher risk category and non-cases move into a lower risk category. The NRI is defined as follows:

(5) $$\begin{array}{l} \text{NRI}=\frac{\#\text{events}\;\text{up}-\#\text{events}\;\text{down}}{\#\text{events}}+\frac{\#\text{non}‒\text{events}\;\text{down}-\#\text{non}‒\text{events}\;\text{up}}{\#\text{non}‒\text{events}}\\ \;=\text{NR}I_{\text{events}}+\text{NR}I_{\text{nonevents}} \end{array}$$

One limitation of the NRI is that it is heavily dependent on the specified risk categories. Pencina et al [14] recommend that this NRI measure should only be used if categories are already established in the field of application and influence care decisions. They also propose a continuous version of the NRI that does not require the pre-definition of risk categories, and can therefore be applied universally [14]. The continuous NRI is estimated by classifying individuals based on the direction of any change in predicted risk, regardless of magnitude.

In estimating the NRI in case-cohort designs it is necessary to consider the effect of censoring the observations that have had an event after *t* years. In the implementation for cohort designs, when using *t* year absolute risk, individuals who had an event after this time are censored and so count in the non-events category. For case-cohort designs this should apply only to cases in the subcohort. Cases outside the subcohort that experience an event after *t* years should be excluded from the calculation. If this exclusion does not occur, then the predicted absolute risk among non-cases will be artificially high, being biased by over-representation of participants who will become cases after *t* years. This modification was not implemented by Ganna et al [4], but would be of less relevance due to the comparatively short follow-up time of the study they consider.

Since the probabilities of movement are evaluated separately for events and non-events, the over-representation of cases in the design does not impact the NRI in the same way as the discrimination metrics and no additional weighting to Equation (5) (or the standard error proposed by Pencina et al [13]) is required. Ganna et al [4] propose a weighted version of the NRI, but this weighting is not necessary for unstratified designs.

An alternative formulation of the NRI for case-cohort designs, in which it is not necessary to exclude the non-subcohort cases that occur after *t* years, is also possible. Under this strategy, the additional individuals are included in the non-event portion of the calculation, and to compensate for the over-representation of cases a weighting of 1/α is applied to the subcohort controls. The non-event portion of the calculation then includes three distinct groups; subcohort and non-subcohort cases that occur after *t* years (both with weight 1) and subcohort controls (with weight 1/α). While we do not present results for this approach, it potentially provides estimates with lower variance due to the inclusion of additional information.

#### Example: Measures of reclassification calculated using case-cohort data

**Figure 3:**
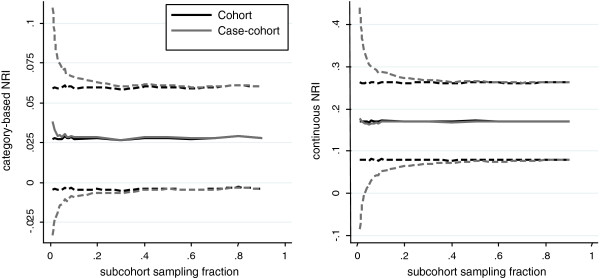
**Category-based NRI (left) and continuous NRI (right) with empirical 95% confidence intervals.** Graphs show the mean over 1000 selections of subcohort at each sampling fraction for case-cohort estimates using the Prentice weighting scheme, and the full cohort estimates.

Figure 3 shows the NRI for 10-year risk predictions using risk models (Table 1) with and without inclusion of HDL-cholesterol. Both the continuous NRI and that based on risk categories of 0-10%, 10-20% and over 20% are shown. The 444 individuals censored before 10 years are not included since we do not know their event status at 10 years. The overall magnitude of the category-based NRI is lower than that of the continuous NRI, since movement from one category to another is less likely to occur than movement defined as any change in predicted risk.

The category-based NRI shows a small amount of bias and larger variability at low subcohort sampling fractions but this reduces as the sampling fraction increases. The continuous NRI appears unbiased even at low sampling fractions. However, as shown in Additional file 1: Figure S4, when the component fractions for cases and controls are considered separately, a small amount of bias is seen in each term. The observed bias can be attributed to bias in the initial 10-year risk predictions obtained using the prediction model derived from case-cohort rather than full cohort data. The increased variance is primarily attributed to the non-event NRI portion of the calculation in Equation 5 since this is calculated using only a subsample of the non-events in the full cohort (Additional file 1: Figure S4). The number of events at 10 years will be the same in the case-cohort sample, as in the full cohort, so this portion of the calculation remains effectively the same.

The analytical SE of the NRI underestimates the empirical SE (Additional file 1: Figure S5). This may be because the analytical SE does not account for potential error in the absolute risk estimates. As shown in Figure 1, the absolute risk predictions show a small amount of bias at low subcohort sampling fractions, and also greater variability. This leads to a larger variability in the resulting NRI estimates than implied by the analytical formula for the SE. This is confirmed by noting that when the case-cohort NRI is estimated using the absolute risks generated from the whole cohort sample (and therefore without bias) the discrepancy does not occur. Instead the empirical SE is greatly reduced and shows good agreement with the analytical estimate. The difference between analytical and empirical SE is also not as marked for the continuous NRI. This is because movement is defined as any change in predicted risk, and so any bias in the absolute risk predictions is less likely to affect the overall proportions of individuals moving up and down than for the category-based measure, where small biases in the overall absolute risk can make large differences to the amount of movement between categories.

### Extended simulations: impact of incidence

In practice, very small subcohort sampling fractions would only be utilised in a study with a low incidence rate. So, our simulation procedure is replicated here for another example with a reduced incidence rate. The reduction in incidence rate is achieved by halving the Weibull rate parameter used to generate the event times in Equation (1). For this second example, the mean 10-year incidence is 0.027, as opposed to the original mean incidence of 0.073.

**Figure 4:**
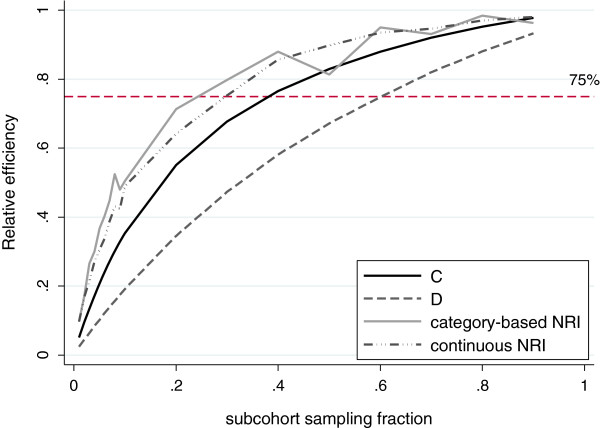
**Relative efficiency of case-cohort methods to the full cohort.** Results are shown for the higher-incidence example, and are calculated using analytical variances for C and D and empirical variances for the NRI.

The results are shown in Table 2a for discrimination measures and Table 2b for the NRI. All results correspond to the mean value over 1000 simulations. Discrimination was evaluated using the full cohort, and for the case-cohort subsample using the standard unweighted measures of discrimination (UW) as well as the weighted versions (W). For the case-cohort estimates the Prentice weighting scheme was used to derive the risk predictions. The relative efficiencies of each of the measures for the higher incidence rate example are also plotted in Figure 4.

**Table 2:** Results of simulation study with alternative incidence rates

**a) Discrimination measures**																
Incidence* 10 years (25 years)	**Sampling fraction**	**% Cases (25 years)**	**Harrell’s C**							**Royston and saubrei’s D**						
			**Full cohort**	**Case-cohort**						**Full cohort**	**Case-cohort**					
				**UW (bias %)**	**W**	**SE**	ESE**	**RE**^**‡**^	**ERE**^**‡‡**^		**UW (bias %)**	**W**	**SE**	ESE**	**RE**^**‡**^	**ERE**^**‡‡**^
0.073 (0.277)	0.03	91	0.674	0.552 (18.1)	0.675	0.018	0.018	0.135	0.126	1.060	0.336 (68.3)	1.099	0.162	0.172	0.064	0.055
	0.1	76		0.591 (12.4)	0.675	0.011	0.011	0.354	0.349		0.568 (46.5)	1.070	0.094	0.092	0.192	0.197
	0.3	52		0.637 (5.5)	0.674	0.008	0.008	0.678	0.672		0.832 (21.4)	1.063	0.060	0.057	0.474	0.479
	0.5	39		0.655 (2.7)	0.674	0.007	0.007	0.831	0.817		0.942 (11.0)	1.060	0.050	0.047	0.671	0.682
	0.9	26		0.672 (0.3)	0.675	0.007	0.006	0.978	0.981		1.046 (1.4)	1.061	0.043	0.040	0.933	0.935
0.027 (0.112)	0.03	76	0.679	0.576 (15.3)	0.681	0.021	0.020	0.283	0.288	1.073	0.491 (54.4)	1.087	0.173	0.167	0.147	0.157
	0.1	49		0.627 (7.6)	0.679	0.014	0.014	0.582	0.580		0.771 (28.1)	1.072	0.109	0.102	0.368	0.4179
	0.3	24		0.662 (2.5)	0.679	0.012	0.011	0.843	0.862		0.970 (9.4)	1.073	0.080	0.075	0.682	0.7407
	0.5	16		0.671 (1.1)	0.679	0.011	0.011	0.926	0.928		1.023 (4.3)	1.069	0.073	0.07	0.823	0.8868
	0.9	10		0.679 (0.1)	0.680	0.011	0.011	0.991	0.997		1.070 (0.5)	1.076	0.068	0.066	0.956	0.9835
**b) NRI**																
Incidence* 10 years (25 years)	**Sampling fraction**	**% Cases (25 years)**	**Category-based NRI**						**Continuous NRI**							
			**Full cohort**	**Case-cohort**					**Full cohort**	**Case-cohort**						
				**NRI**	**SE**	ESE**	**RE**^**‡**^	**ERE**^**‡‡**^		**NRI**	**SE**	ESE**	**RE**^**‡**^	**ERE**^**‡‡**^		
0.073 (0.277)	0.03	97	0.028	0.029	0.025	0.033	0.414	0.265	0.170	0.166	0.086	0.095	0.296	0.214		
	0.1	86		0.028	0.019	0.023	0.713	0.506		0.170	0.060	0.069	0.602	0.490		
	0.3	76		0.027	0.017	0.018	0.906	0.798		0.169	0.050	0.051	0.854	0.753		
	0.5	52		0.028	0.017	0.018	0.960	0.814		0.171	0.048	0.050	0.932	0.898		
	0.9	26		0.028	0.016	0.018	0.997	0.963		0.170	0.047	0.046	0.992	0.981		
0.027 (0.112)	0.03	91	0.011	0.015	0.014	0.022	0.571	0.308	0.156	0.154	0.106	0.114	0.561	0.456		
	0.1	66		0.012	0.012	0.015	0.813	0.603		0.160	0.087	0.096	0.822	0.730		
	0.3	49		0.010	0.011	0.012	0.954	0.965		0.156	0.081	0.080	0.947	0.903		
	0.5	24		0.011	0.011	0.013	0.990	0.933		0.160	0.080	0.083	0.976	0.959		
	0.9	10		0.011	0.011	0.012	1.008	0.944		0.155	0.079	0.076	0.997	0.978		

Results are shown for the full cohort, the case-cohort subsample with Prentice weights using the standard unweighted measures of discrimination (UW) as well as the weighted versions (W). For the weighted estimates we also show the analytical and empirical SE and relative efficiency.

* Incidence from Kaplan-Meier.

** Empirical standard error.

^‡^ Relative efficiency calculated as the ratio of analytical variances (full cohort/case-cohort).

^‡‡^ Empirical relative efficiency calculated as the ratio of empirical variances (full cohort/case-cohort).

As described previously, the unweighted discrimination measures are biased. For a given sampling fraction the bias is less pronounced for the lower incidence rate as the over-representation of cases is less extreme. The relative efficiency for discrimination and the NRI is also more favourable for the lower incidence rate. As would be expected, as the incidence rate increases, a larger subcohort sampling fraction is required in order to maintain the same efficiency compared to the full cohort.

## Discussion

We have described methods to derive and assess the predictive ability of risk models using case-cohort data. The derived risk prediction models were shown to give slightly elevated and more variable risk predictions at small subcohort sampling fractions, but otherwise were comparable with those which might be achieved in full cohort data. We have demonstrated that failure to account for case-cohort sampling can lead to biased results when assessing predictive ability, and have adapted three measures of predictive ability that then give results comparable to that which would be obtained with full cohort data. The following Stata [24] commands to implement these methods are available at http://www.phpc.cam.ac.uk/ceu/research/erfc/stata: ‘mvmetaipd’, which can be used to fit prediction models, and ‘predaddc’, ‘predaddd’ and ‘predstat’ which can be used to estimate the C-index, D measure and NRI respectively. These commands can be applied with cohort data, case-cohort data, and single or multiple studies according to the options selected.

For both Harrell’s C and Royston and Sauerbrei’s D the standard methods return lower estimates when applied to case-cohort designs than when applied to the full cohort data set. For Harrell’s C, this bias arises because of the over-representation of case-case pairs, which are less likely to be concordant than case–control pairs. For Royston and Sauerbrei’s D, bias arises as the overall risk is inflated due to the inclusion of extra cases, leading to reduced separation between those at low and high predicated risk. The magnitude of the bias is dependent on the subcohort sampling fraction, with the bias increasing as the subcohort sampling fraction decreases (and hence the enrichment of cases in the data set increases).

Applying a weighted version of these discrimination measures provides estimates similar to that which would have been expected in the full cohort. This weighting should also be applied when assessing the changes in the C-index or D on addition of a new marker. Failure to weight the measures of predictive ability for each model could lead to an underestimation of the change in predictive ability. A small amount of bias remains with the weighted version of D at low subcohort sampling fractions, which may be due to the fact it is estimated as the coefficient from a weighted Cox model. When applying such weighted Cox models in the initial risk model derivation, we found similar slight bias.

Another important consideration is the possibility of overfitting at low subcohort sampling fractions, leading to inflated discrimination metrics. In the results provided, the bias seen at low subcohort sampling fractions is greater for D than for the C-index. If this bias was due to overfitting in estimation of the risk model then we would expect this effect to be observed for both C and D and so we conclude that this bias is introduced due to the procedure for estimating D rather than overfitting. In assessing bias our gold standard was evaluation of the full-cohort model in the full cohort. Hence, bias can arise both from differences in the fitted model (possibly overfitting) and in the calculation of prediction metrics. A reviewer has correctly pointed out that an additional comparison, which would allow isolation of error due to differences in the fitted model, would be evaluation of the case-cohort model in the full cohort; we suggest that yet another comparison would be evaluation in an independent cohort. However, our results show that the weighted evaluations agree closely with our gold standard; there is little bias to explain, suggesting that our gold standard was adequate in these large samples. Although it is not of concern in our example due to the relatively large sample size of the original cohort, the possibility of overfitting is an important consideration when analysing smaller case-cohort studies with low subcohort sampling fractions.

For the NRI, no adaptation to the standard calculations is required but individuals who experienced an event after the chosen cut-off at *t* years must be excluded from the calculation unless they belong to the subcohort. Application of both the category-based and continuous NRI was considered, with less bias observed at small sampling fractions for the continuous NRI.

The results of the simulation studies at two choices of incidence rate provide guidance on the efficiency of the described case-cohort weighted methods. For data with the same properties (i.e. full cohort size, follow-up and incidence) as the Reykjavik study, the relative efficiency of the prediction metrics for the case-cohort design was close to that of the full cohort (based on 75% efficiency, Figure 4) provided that the subcohort sampling fraction was above 40% for the C-index, 60% for the D measure or 30% for the NRI. This is greater than the subcohort sampling fraction, of around 10%, found by Onland-Moret et al [3] to be necessary to achieve reasonable accuracy and precision in coefficients alone. For the C-index and D measure the generated analytical SEs were very similar to the corresponding empirical estimates. However this was not the case for the NRI, for which the analytical SE was lower. The NRI relies on absolute risk predictions, which themselves are subject to some bias and extra variability when estimated using case-cohort data. Small changes in absolute risk predictions can greatly affect risk categorisation and we believe this to be the origin of the extra empirical variation. This is not as relevant for D and the C-index, which rely only on comparing ranks of the linear predictor, and is less of an issue with the continuous NRI which does not rely on categorising predicted absolute risk. We conclude that analytical SEs may not be appropriate for the NRI calculated from case-cohort designs and recommend that SEs should instead be computed by bootstrapping. The relative efficiency for all measures investigated is shown to be more favourable for the lower incidence rate.

Extension to the multi-study setting is also possible, using the methods described elsewhere [25,26]. This applies when combing multiple studies each with a case-cohort design, but also when combining studies of different designs. The risk prediction model can be derived using a (weighted) CPH model stratified by study and, if applicable, sex, producing one set of coefficients over all studies. If the Breslow weighting method is applied, different subcohort sampling fractions may be specified for each study. Similarly, in order to estimate absolute risks the weighting applied to the Breslow estimator may vary by study. To pool study-specific estimates of predictive ability in the case of full cohort data, it has been suggested to weight by the number of events in each study [25,26]. This choice is also intuitively appealing for case-cohort designs, although other methods may be appropriate if pooling across case-cohort studies with a large amount of heterogeneity in the subcohort sampling fraction.

The presented simulations are based on real data, giving the advantage of representing the properties (e.g. distributions and associations between covariates) of typical epidemiological studies. However, in order to provide results that are generalisable to wider application, two choices of incidence rate were considered. Another factor that affects the observed results is the size of the initial cohort study. For a larger starting cohort size, the results at a given subcohort sampling fraction will have lower variance.

The C-index is slightly sensitive to random censoring [27], and the presented NRI calculation excludes individuals censored before the time point of interest. Our focus was to contrast results using the proposed measures for case-cohort data to those which would have been seen in original (censored) cohort data, and we have shown the two to be comparable. They remain comparable for two different incidence rates (which induce different degrees of censoring). The implication of this is that the estimates of predictive ability made using case-cohort data will be similarly subject to censoring induced bias as are full cohort estimates.

We have not considered competing risks in our proposed methods and simulations, which will be important for example when predicting lifetime risks. If competing risks are included when estimating risk then further adaptation of measures of discrimination are necessary [28]. Likewise, if non-proportional hazards are modelled, if time dependant covariates are included, or if age rather than time from study entry is used as the time scale, then the ranking of $\hat{\beta }x_{i}$ can change with time. Calculation of measures of discrimination in such instances is a matter for further research. Competing risks are unlikely to make a substantial difference when making 10-year CHD risk predictions for our example participants, who are mostly under the age of 60 (Table 1).

## Conclusions

In summary, this paper provides a thorough description of the issues related to deriving and assessing predictive ability in case-cohort studies. With the increasing use of case-cohort designs to answer questions relating to risk prediction, the methods presented in this paper will enable suitable measures of predictive ability to be computed and compared with studies of other designs.

## Competing interests

The authors declare that they have no competing interests.

## Authors’ contributions

JS devised the adapted statistical methods presented, designed and then carried out all analyses, and drafted the manuscript. LP conceived of the study, assisted with adaptation of the presented methods, was instrumental in design of the simulation study and interpretation of results, and helped to draft the manuscript. ST, IW and TA participated in design and interpretation of the simulation study and development of the presented method adaptations and provided critical review of the text. TA also represents the Reykjavik study, and provided data on which simulation were based. All authors read and approved the final manuscript.

## Pre-publication history

The pre-publication history for this paper can be accessed here:

http://www.biomedcentral.com/1471-2288/13/113/prepub

## Supplementary Material

Additional file 1: Figure S1 Mean error in 10-year cumulative baseline survival. **Figure S2.** Error in log hazard ratios. **Figure S3.** Empirical and mean analytical SE for the C-index and D measure: full cohort and case-cohort estimates. **Figure S4.** Category-based and continuous NRI for events and nonevents:full cohort and case-cohort estimates. **Figure S5.** Empirical and mean analytical standard errors for category-based and continuous NRI: full cohort and case-cohort estimates. Click here for file
