# Supplementary material for: Derivation and assessment of risk prediction models using case-cohort data
Source: BMC Med Res Methodol. 2013 Sep 13;13:113. doi: 10.1186/1471-2288-13-113 (PMC3848813; doi:10.1186/1471-2288-13-113)

**Supplementary Material to accompany:**

# Derivation and assessment of risk prediction models using case-cohort data

Jean Sanderson[1], Simon G Thompson[1], Ian R. White[2], Thor Aspelund[3,4], Lisa Pennells[1]

[1] Department of Public Health and Primary Care, University of Cambridge, UK

[2] MRC Biostatistics Unit, Cambridge, UK

[3] University of Iceland

[4] Icelandic Heart Association

**Supplementary Figure 1: Mean error in 10-year cumulative baseline survival**

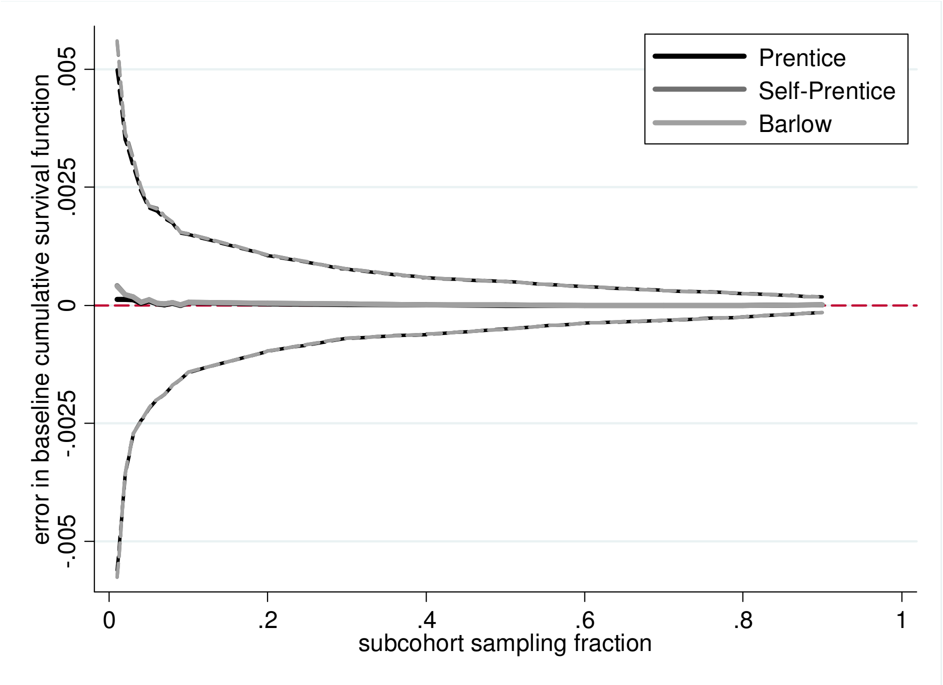


Figure shows the difference between cumulative baseline survival obtained using a prediction model derived on the case-cohort set, and that obtained using the model derived on the full cohort. Solid lines show mean error, and dashed lines show empirical upper and lower fifth percentiles, averaged over all individuals in the case-cohort set, and averaged over simulations, for three case-cohort weighting methods.

**Supplementary Figure 2: Error in log hazard ratios**

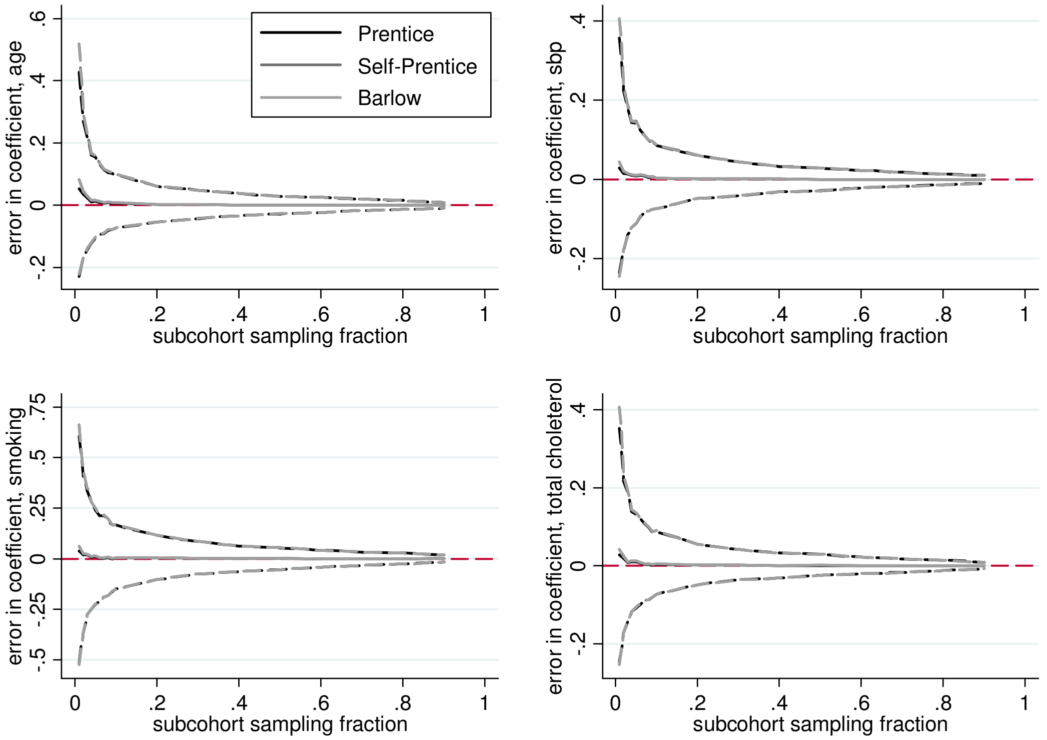


Figure shows the difference between log hazard ratios obtained using a model derived on the case-cohort set, and that obtained using the model derived on the full cohort. Solid lines show mean error, and dashed lines show empirical upper and lower fifth percentiles, averaged over all individuals in the case-cohort set, and averaged over simulations, for three case-cohort weighting methods.

**Supplementary Figure 3: Empirical and mean analytical SE for the C-index and D measure: full cohort and case-cohort estimates.**

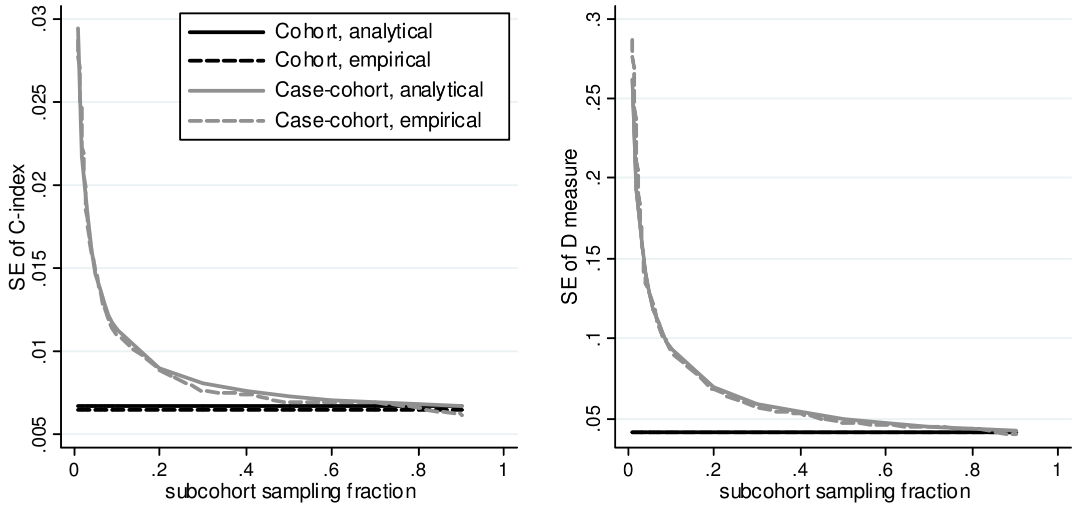

**Supplementary Figure 4: Category-based and continuous NRI for events and non-events: full cohort and case-cohort estimates**

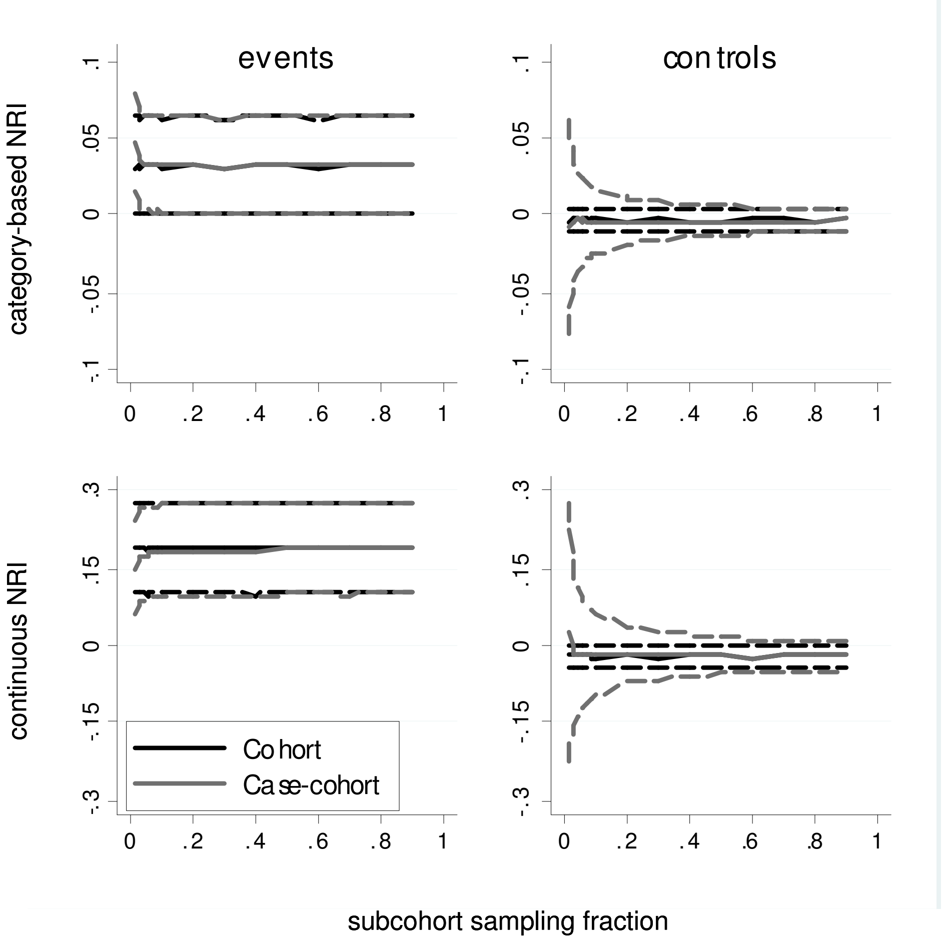


Dotted lines indicate empirical 95% CI

**Supplementary Figure 5: Empirical and mean analytical standard errors for category-based and continuous NRI: full cohort and case-cohort estimates.**

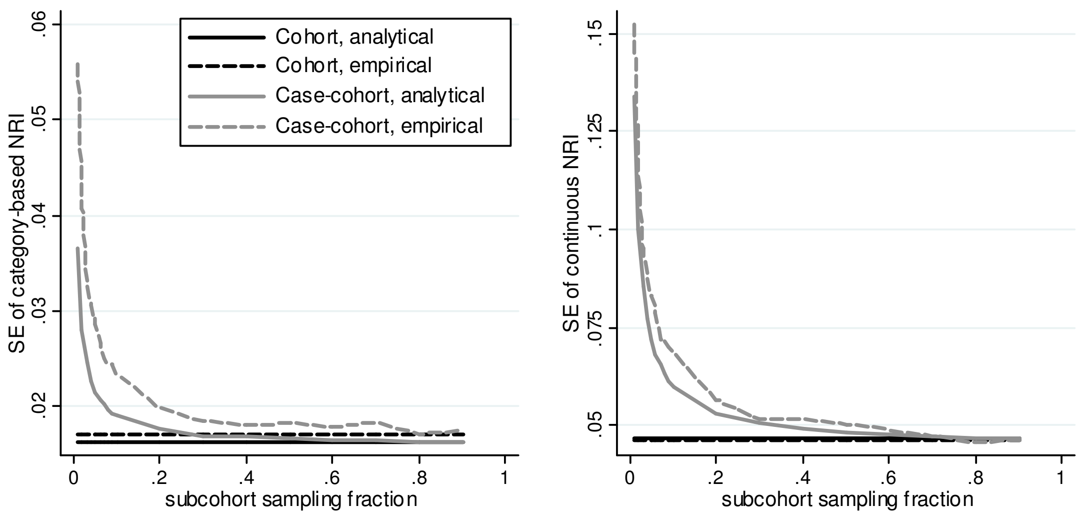

Supplement: Additional file 1: Figure S1 — Mean error in 10-year cumulative baseline survival. Figure S2. Error in log hazard ratios. Figure S3. Empirical and mean analytical SE for the C-index and D measure: full cohort and case-cohort estimates. Figure S4. Category-based and continuous NRI for events and nonevents:full cohort and case-cohort estimates. Figure S5. Empirical and mean analytical standard errors for category-based and continuous NRI: full cohort and case-cohort estimates. [file 1471-2288-13-113-S1.pdf]
